# Retraction: analysis of the TCR alpha and beta chain CDR3 spectratypes in the peripheral blood of patients with Systemic Lupus Erythematosus

**DOI:** 10.1186/1740-2557-5-5

**Published:** 2008-08-11

**Authors:** Wei Luo, Li Ma, Qian Wen, Xin-Sheng Yao, Na Wang, Hong-Yun Zou, Ming-Qian Zhou, Ying Lin, Zhen-Qiang Wu, Xiao-Wei He, Ju-Fang Wang, Xiao-Ning Wang

**Affiliations:** 1 Institute of Molecular Immunology, Southern Medical University, Guangzhou, 510515, PR China; 2 College of Biosciences & Bioengineering, South China University of Technology, Guangzhou, 510641, PR China; 3 Department of Dermatology & Rheumatology, Nan fang Hospital, Guangzhou, 510515, PR China

BioMed Central is publishing a retraction of this article [1] as it was published in error. BioMed Central apologize to the authors for the error and any inconvenience caused. An apology is also extended to the readers.
